# News and Coming Events

**Published:** 1909-02-13

**Authors:** 


### News and Coming Events.

Dr. J. A. Haran, Medical Officer of the East Africa Protectorate, has become Medical Officer of Health for Mombasa.

The Lord President of the Council has appointed the Hon. Mrs. Charles Egerton to be a member of the Central Midwives Board in the place of Miss Jane Wilson, recently resigned.

According to a *Reuter's* telegram from Heidelburg dated February 5 a gift of 130,000 marks (£6,500) has been made to Heidelburg University for the foundation of a radium institute. The work of the institute will begin at Easter.

The Ceylon Opium Bill was withdrawn on February 3, on the second reading, pending the publication of its report by the Shanghai Conference. It is hoped in official quarters to reintroduce the Bill at the end of the present year. Meanwhile, we learn that the Bill prohibiting the importation of opium into the United States, except for medicinal purposes, has been passed by both Houses of the United States Congress.

Mr. Sinclair White, F.R.C.S., surgeon to the Royal Infirmary, Sheffield, has been elected a corresponding member of the Surgical Society of Paris.

The Princess of Wales has consented to open, on Monday, February 22, the new ward for children at the Great Northern Central Hospital, Holloway, and will receive purses containing subscriptions towards the cost of construction and equipment.

The Royal Waterloo Hospital for Children and Women, Waterloo Road, London, S.E., announces that its appeal last year for old Christmas cards resulted in 30,000 cards being sold for £31 10s., and a cot thereby being founded. Miss Dorothy Nicholson, 4 Sloane Court, S.W., is in charge of this scheme.

The first number of *Slainte* (Celtic for "Health"), the official organ of the Women's National Health Association, has lately been published in Dublin. The Countess of Aberdeen is acting as its editor, and the publication will contain articles on health conditions in Ireland, and notes by Lady Aberdeen on the campaign against tuberculosis in Ireland.

His Excellency the Lord Lieutenant of Ireland, the Earl of Aberdeen, has accepted the invitation of the President and Fellows of the Royal College of Surgeons in Ireland to the annual college dinner on Saturday, February 20.

The annual oration before the Hunterian Society was delivered by Dr. W. Rawes (medical superintendent of St. Luke's Hospital) on Wednesday, February 10, at the London Institution, Finsbury Circus. The title of his address was "Psychiatry: A Retrospect."

Mr. Newstead and Mr. Prout, members of the expedition sent to Jamaica by the Liverpool School of Tropical Medicine, left Kingston, Jamaica, on January 28, to return to England. Their services are stated by the *Times* Jamaica correspondent to have proved of great value to Jamaica, especially their advice regarding the prevention of diseases. Dr. Hanley is remaining at Kingston.

The death is reported of Mr. Thomas Joseph Patrick Hartigan, F.R.C.S., Eng., surgeon-pathologist and medical officer in charge of the Light Department of the Hospital for Diseases of the Skin, Blackfriars, and dermatologist to the Alexandra Hospital, Bloomsbury. Mr. Hartigan was the inventor of a new form of radium applicator. He qualified in 1898, and became a Fellow of the Royal College of Surgeons in the following year.

The Hunterian Oration of the Royal College of Surgeons of England will be delivered in the theatre of the College by Mr. Henry Morris, the President, on Monday, February 15, at 4 p.m. Fellows and Members of the College who desire to be present are asked to apply for tickets to the Secretary, Mr. S. Forrest Cowell. The College will be honoured on this occasion by the presence of the Prince and Princess of Wales.

The Prince and Princess of Wales paid a surprise visit to St. Mary's Hospital, Paddington, on the afternoon of Monday, February 8. They were conducted over the hospital by the Secretary and the Medical Superintendent. The Prince and Princess inspected the wards in the old hospital, and visited the new operating theatre and the empty wards of the Clarence wing, which they regretted to see still unoccupied. On the following afternoon the Prince and Princess of Wales paid a visit to St. Bartholomew's Hospital, and were shown round many of the wards and other parts of the buildings by the Treasurer (Lord Sandhurst). The Prince of Wales is President of both hospitals.

Surgeon-General Thomas Tarrant, C.B., who died on February 3, was born in 1830. At the age of twenty-four he proceeded to the Crimea, where he served at the siege of Sebastopol, receiving the British and Turkish medals. He next took part in the Mutiny campaign and was present at the battle of Cawnpore, the action of Kalee Nuddee, and the affair of Kunkur. In 1866 he reached the rank of surgeon and in 1873 that of surgeon-major. In 1878 he served as Senior Medical Officer in Wood's force in South Africa, while in the next year he took part in the Zulu war. For his services he was mentioned in despatches and received the medal and clasp. In 1883 he reached the rank of Deputy Surgeon-General, and six years later that of Surgeon-General, retiring from the service twelve months afterwards. In 1903 he was appointed Honorary Physician to the King, and in 1907 was made a C.B.

The Prince of Wales has increased his subscription to St. George's Hospital from twenty guineas to fifty pounds per annum.

The annual meeting of the Royal Free Hospital, Gray's Inn Road, will be held at the Mansion House at 3 p.m., on Wednesday, March 10, the Lord Mayor presiding.

Lord Clifden, deputy-chairman of the committee of the Mount Vernon Hospital for Consumption and Diseases of the Chest, will preside at a festival dinner to be held in aid of the funds of the hospital on May 13 next.

A series of Sunday evening concerts, arranged by Chevalier Arrigo Bocchi, a director of Ashton's Royal Agency, will be given at the Coronet Theatre, lent by Mr. Robert Arthur, in aid of the West London Hospital.

The special committee appointed by the Wandsworth Borough Council report that an order has been received from the Charity Commissioners authorising the council to appeal against the Weir Hospital Charity Scheme.

Lieut.-Colonel J. W. T. Gilbert, V.D., Royal Army Medical Corps (Territorial), has received his Majesty's permission to accept and wear the silver medal of the Order of Orange-Nassau, conferred upon him by the Queen of the Netherlands.

The second international course of legal psychology and psychiatry will be held at Giessen, April 13 to 18, 1909. The course will be under the direction of Professor Sommer, who will have the co-operation of Professors Mittermaier and Dannemann of Giessen, and Professor Aschaffenburg of Cologne. All communications should be addressed to Professor Dr. Sommer, University of Giessen.

The death occurred at Godalming on February 1 of Dr. William Parson, an ex-mayor of the borough, at the age of seventy-four. Dr. Parson was medical officer of health at Godalming for twenty-seven years, and was an ex-master of the Apothecaries Company. He was made a magistrate soon after the commission was granted to the borough of Godalming in 1879. He qualified at the Royal College of Surgeons in 1855. He was at one time house surgeon of the Hospital for Sick Children, Great Ormond Street.

The English Conjoint Board has added King's College School, Wimbledon, and Whitgift School, Croydon, to the institutions recognised for instruction in chemistry and physics, and the Portsmouth Municipal Technical Institute to the institutions recognised for instruction in biology. The Medico-Chirurgical College, Philadelphia, has also been added to the institutions at which the curriculum of professional study required for the diplomas of the Royal Colleges may be pursued, and whose graduates may be admitted to the final examination on production of the required certificates of study.

Sir Arthur Vernon Macan, of Merrion Square, Dublin, a former President of the Royal College of Physicians in Ireland, and President of the Obstetrical Section of the International Medical Congress and the Royal Academy of Medicine, and President of the British Gynæcological Society, who died on September 26, aged sixty-five, left personal estate in the United Kingdom valued at £25,924. The testator left his midwifery instruments and objects for the teaching of midwifery to Trinity College, Dublin, and his French and German medical books and his English books on midwifery to the Royal College of Physicians, Ireland.

